# Effect of the diet type and temperature on the *C. elegans* transcriptome

**DOI:** 10.18632/oncotarget.23563

**Published:** 2017-12-21

**Authors:** Eva Gómez-Orte, Eric Cornes, Angelina Zheleva, Beatriz Sáenz-Narciso, María de Toro, María Iñiguez, Rosario López, Juan-Félix San-Juan, Begoña Ezcurra, Begoña Sacristán, Adolfo Sánchez-Blanco, Julián Cerón, Juan Cabello

**Affiliations:** ^1^ CIBIR (Center for Biomedical Research of La Rioja), Logroño, 26006 La Rioja, Spain; ^2^ Bellvitge Biomedical Research Institute - IDIBELL, L’Hospitalet de Llobregat, 08908 Barcelona, Spain; ^3^ University of La Rioja, Logroño, 26006 La Rioja, Spain; ^4^ Hospital San Pedro, Logroño, 26006 La Rioja, Spain; ^5^ Department of Biology, University of Hartford, West Hartford, 06117 CT, USA; ^6^ Present address: Institut Pasteur, 75015 Paris, France

**Keywords:** transcriptomic analysis, physiological response, growth temperature, diet, caenorhabditis elegans, Gerotarget

## Abstract

The transcriptomes of model organisms have been defined under specific laboratory growth conditions. The standard protocol for *Caenorhabditis elegans* growth and maintenance is 20°C on an *Escherichia coli* diet. Temperatures ranging from 15°C to 25°C or feeding with other species of bacteria are considered physiological conditions, but the effect of these conditions on the worm transcriptome has not been well characterized. Here, we compare the global gene expression profile for the reference *Caenorhabditis elegans* strain (N2) grown at 15°C, 20°C, and 25°C on two different diets, *Escherichia coli* and *Bacillus subtilis*. When *C. elegans* were fed *E. coli* and the growth temperature was increased, we observed an enhancement of defense response pathways and down-regulation of genes associated with metabolic functions. However, when *C. elegans* were fed B. subtilis and the growth temperature was increased, the nematodes exhibited a decrease in defense response pathways and an enhancement of expression of genes associated with metabolic functions. Our results show that *C. elegans* undergo significant metabolic and defense response changes when the maintenance temperature fluctuates within the physiological range and that the degree of pathogenicity of the bacterial diet can further alter the worm transcriptome.

## INTRODUCTION

The reproducibility of results obtained in studies using model organisms highly depends on uniformity of growing conditions. The nematode *Caenorhabditis elegans* is widely used as a model organism in biology. The vast majority of laboratories use the *C. elegans* wild type N2 strain as reference, which was isolated by L. N. Staniland in Bristol (UK) in 1959 [1]. To minimize experimental variation, *C. elegans* growth and maintenance protocols are highly standardized. The canonical conditions for growing *C. elegans* in the lab are 20°C temperature and *E. coli* OP50 bacteria as diet [2]. 15°C–25°C temperature maintenance is however considered physiological. Temperatures outside this range are not considered physiological and have a harmful effect on the development and physiology of the worm (http://www.wormbook.org). Using physiological temperatures above and below 20°C is a common practice when performing studies with *C. elegans* in the lab and it is especially important when analyzing thermosensitive mutants or when performing RNAi assays. Temperature fluctuations are known to have a major effect on parameters that reflect the physiology of the worm such as developmental time or longevity. For instance, at 25°C, laboratory maintained wild type *C. elegans* complete the life cycle in 2.5 days, whereas at 15°C worm development slows down and the life cycle extends up to 5 days. Similarly, the mean life span of *C. elegans* kept at 16°C or at 24°C increases 60% or decreases 50%, respectively relative to worms maintained at 20°C [3]. Thus, *C. elegans* studies performed at temperatures within the 15–25^o^C physiological range may elicit different biological responses by the worm, yet results and conclusions from studies at different temperatures are often compared.

The use of *E. coli* OP50 as *C. elegans* lab diet responds to historical reasons [2]. *E. coli* was a commonly studied bacterium at the time *C. elegans* was being developed as a model organism. Since *C. elegans* is a bacteriovore, *E. coli* became a very convenient food source to maintain worms and was therefore adopted as the standard *C. elegans* lab diet. In the wild, *C. elegans* feeds on soil and rotting vegetable bacteria and *E. coli* is not a bacterium type that *C. elegans* would typically encounter [4–5].

*Bacillus subtilis*, a common soil bacterium, has been used as an alternative *C. elegans* diet in a number of *C. elegans* studies [6–11]. *B. subtilis* has a similar caloric and nutrition content as *E. coli* [11]. High-energy demanding metabolic processes such as egg production and developmental growth are comparable when worms are fed *E. coli* or *B. subtilis* [11]. Interestingly, the mean life span of *E. coli* fed worms maintained at 20°C has been shown to be around 17 days, compared to 27 days for *B. subtilis* fed worms maintained at the same temperature [6, 8]. In addition, these diets appear to induce death to the worms through different causes [8]. In fact, genetic and environmental interventions that alter *C. elegans* longevity by affecting metabolism, immune system or stress resistance, do not always lead to same effects when worms are fed *E. coli* or *B. subtilis* [11].

In this report we sought to evaluate the impact that different types of commonly used laboratory conditions can exert on worm transcriptome and physiology and therefore their influence in experimental results. To do this, we analyzed the transcriptomic profiles of adult *C. elegans* N2 nematodes grown at 15°C, 20°C and 25°C when fed two different bacterial diets, *E. coli* and *B. subtilis*. By comparing gene expression signatures obtained under these maintenance conditions, we found that diet differences had a strong influence on the transcriptomic pattern of the worm. Interestingly, the effect of temperature on the transcriptome of *E. coli* fed worms was opposed to the effect of the same temperature on *B. subtilis* fed worms. For instance, analysis of biological process enrichment on the differentially expressed genes revealed that *E. coli* fed worms maintained at 25°C led to an up-regulation of genes involved in defense response and down-regulation of genes involved in metabolism. In contrast, maintaining *B. subtilis* fed worms at 25°C caused an up-regulation of genes involved in metabolic processes and a down-regulation of defense response pathways.

In summary, our work highlights major *C. elegans* transcriptomic response differences when animals are maintained at three physiologically accepted temperatures, 15°C, 20°C and 25°C, and fed two different diets, *E. coli* and *B. subtilis*. Gene expression differences revealed significant metabolic adaptation mechanisms of the worm when confronted with physiologically higher or lower temperatures. Moreover up-regulation of defense response genes was observed when worms were fed *B. subtilis*, regardless of the temperature at which worms were kept. Altogether, our data suggest that *C. elegans* undergo significant metabolic and defense response changes when maintenance temperature fluctuates within the physiologically accepted experimental range.

## RESULTS

### Transcriptomic analyses reveal diet and temperature dependent gene expression

**Figure 1:**
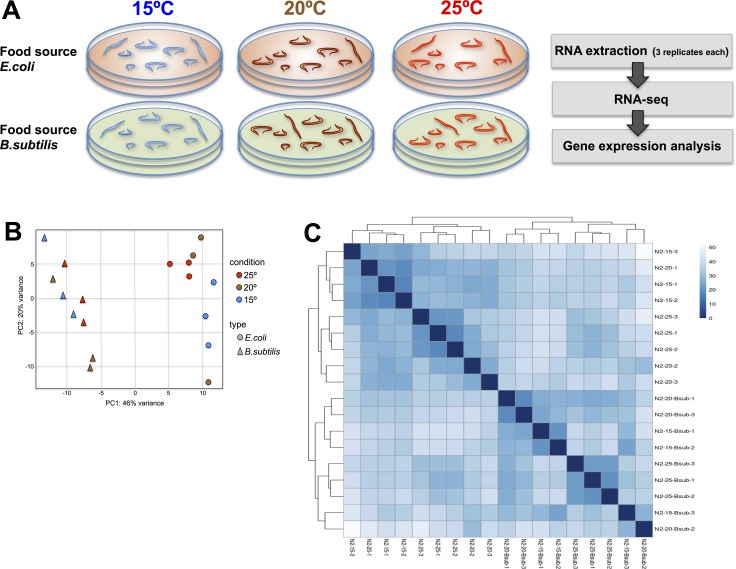
Transcriptional effect of diet and temperature on *C.elegans* (**A**) Strategy for transcriptional profiling. Synchronous 1 day old adult worms maintained at 15°C, 20°C or 25°C and fed either *E. coli* or *B. subtilis* were collected. RNA extraction and deep-sequencing was performed using commercial kits and Illumina platform using three biological replicates per condition. Analysis on differential gene expression data was performed as described in Material and Methods. (**B**) Principal Component Analysis (PCA) plot based on DESeq2 regularized log2 transformation (rld) data. The 18 samples shown in the 2D plane spanned by their first two principal components. Temperature growing condition (color) and food type (plotting symbol) are specified. PC1 shows a clear separation between transcriptomes of worms maintained on the different diets while a modest separation is observed for worms maintained on different temperatures. (**C**) Sample-to-sample distance heatmap showing the Euclidean distances (calculated from the rld data) between the worm samples. Upper and left-side dendrograms show samples grouped by diet type.

To examine the gene expression pattern of WT worms grown at different physiological temperatures we performed global mRNA-seq by deep sequencing using Illumina technology. Illumina platforms have been successfully used in *C. elegans* for both, genome sequencing [26–27] as well as transcriptomic analysis [28]. Synchronized populations of adult worms were maintained at either 15°C, 20°C or 25°C and in two different diets, *E. coli* OP50 and *B. subtilis* PY79 (Figure 1A). Worms were kept on their respective experimental conditions for at least 3 generations prior to performing the mRNA-seq experiments (see Material and Methods). Once the worms were collected, mRNA was purified from the samples and processed for sequencing. Raw sequence data generated in this study are available at the Gene Expression Omnibus (GEO) data repository (Accession number GSE101524). mRNA reads were aligned to the *C. elegans* N2 reference genome (release WB235.85) (http://www.ensembl.org/Caenorhabditis_elegans/Info/Index). After read count on gene features (FeatureCounts software, http://subread.sourceforge.net/), quantitative analysis of differential expression between conditions was performed by following the SARTools pipeline (https://github.com/PF2-pasteur-fr/SARTools) (see Materials and Methods for details).

Gene expression data were normalized by a negative binomial distribution model by using DESeq2 and EdgeR implementations and compared in pairs to analyze the effect of the two variables of this study: diet (*E. coli* and *B. subtilis*) and growth temperature (15°C, 20°C, and 25°C). Common up- and down-regulated genes determined by both methodologies were used for annotation, GO and KEGG analyses.

We used two different clustering methods to analyze transcriptomic similarities between worms maintained on the different temperature and diet conditions. Interestingly, we found a strong transcriptomic correlation for worms fed the same diet independently of the temperature in which they were maintained. The diet correlation was significantly stronger than the correlation observed for worms maintained at the same temperature (Figure 1B and 1C). These data indicate that diet type has a greater effect than physiological temperature variations on the worm transcriptome.

### Experimental validation of mRNA-sequencing results

**Figure 2:**
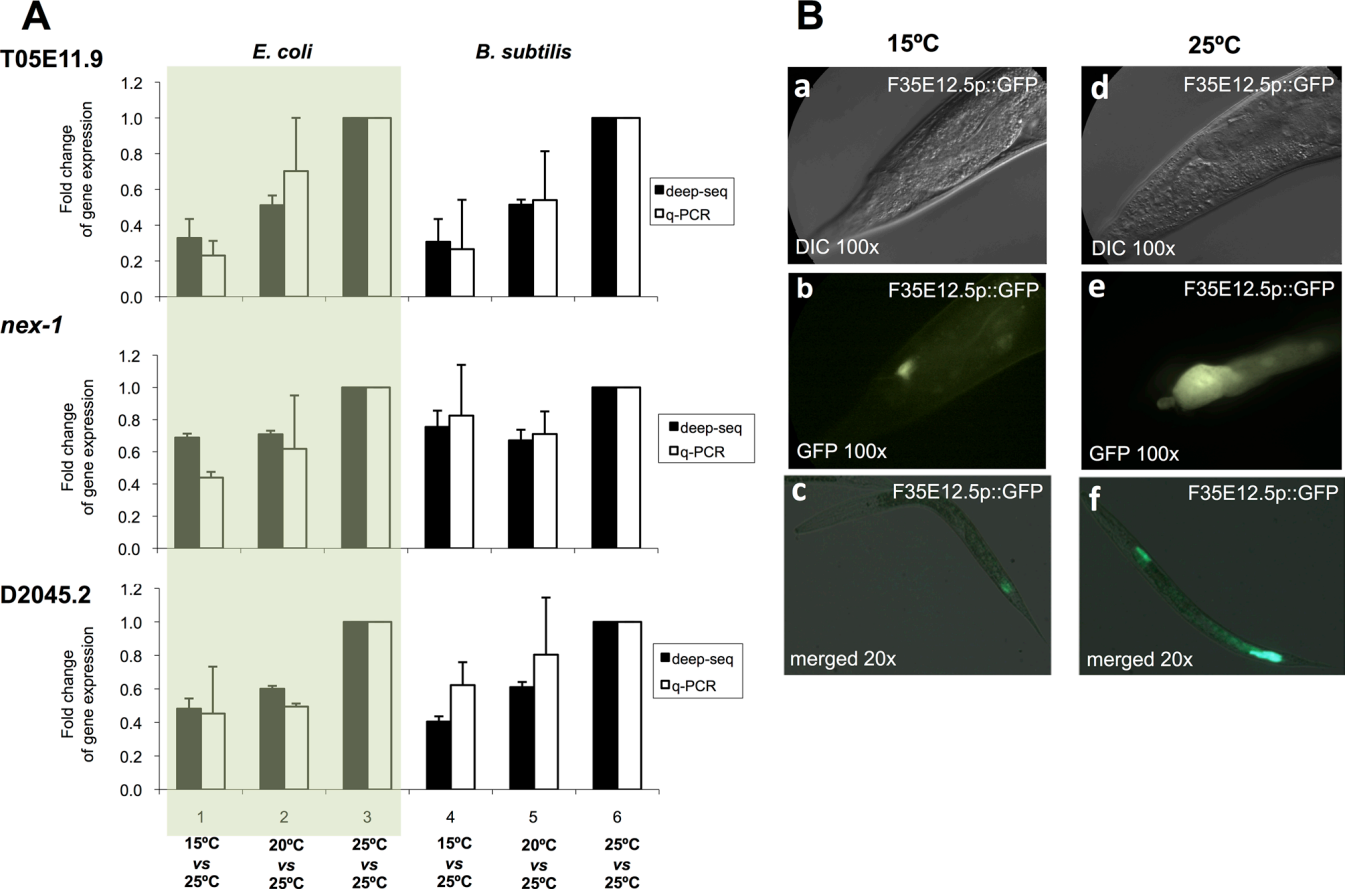
Validation of mRNA-sequencing results (**A**) Gene expression quantification by deep-sequencing (dark bars) and RT-qPCR (light bars) for three genes, T05E11.9, *nex-1*, and D2045.2, which display a significant differential expression at different physiological temperatures. Bars represent fold change for gene expression when worms were maintained at 15°C versus 25°C, or at 20°C versus 25°C. 25°C versus 25°C is used as control. Experiments 1, 2, 3 were performed using an *E. coli* diet and experiments 4, 5, 6 were performed using a *B. subtilis* diet. Each bar represents 3 biological replicates. Results are expressed as the mean ± SEM. (**B**) GFP expression of transgenic worms carrying an integrated GFP transcriptional reporter for F35E12.5 at 15°C (a, b, c) and at 25°C (d, e, f). Detail of the worm tail when maintained at 15°C using Nomarski optics (a) or fluorescence (b) and when maintained at 25°C using Nomarski optics (d) or fluorescence (e). Merged Nomarski/fluorescence channels showing entire worm bodies of worms maintained at 15°C (c) or at 25°C (f).

To validate the mRNA deep sequencing results, we selected three representative genes with significant differential expression when worms were maintained at 15°C or at 25°C (T05E11.9, *nex-1* and D2045.2) and tested the levels of expression of these genes by quantitative Real Time PCR (RT-qPCR) [29]. In all cases we confirmed the direction and magnitude of the expression change observed by deep sequencing (Figure 2A).

To further validate our results, we obtained a transcriptional GFP reporter strain for the defense response gene *irg-5* (F35E12.5) that showed increased expression when worms were maintained at 25°C relative to worms maintained at 15°C. We observed these worms under the microscope at both temperatures and as predicted by the mRNA deep sequencing results, GFP levels were higher for worms maintained at 25°C than for worms maintained at 15°C (Figure 2B).

### Transcriptomic effect of growth temperature variations when *C. elegans* are fed the *E. coli* or the *B. subtilis* diet

**Figure 3:**
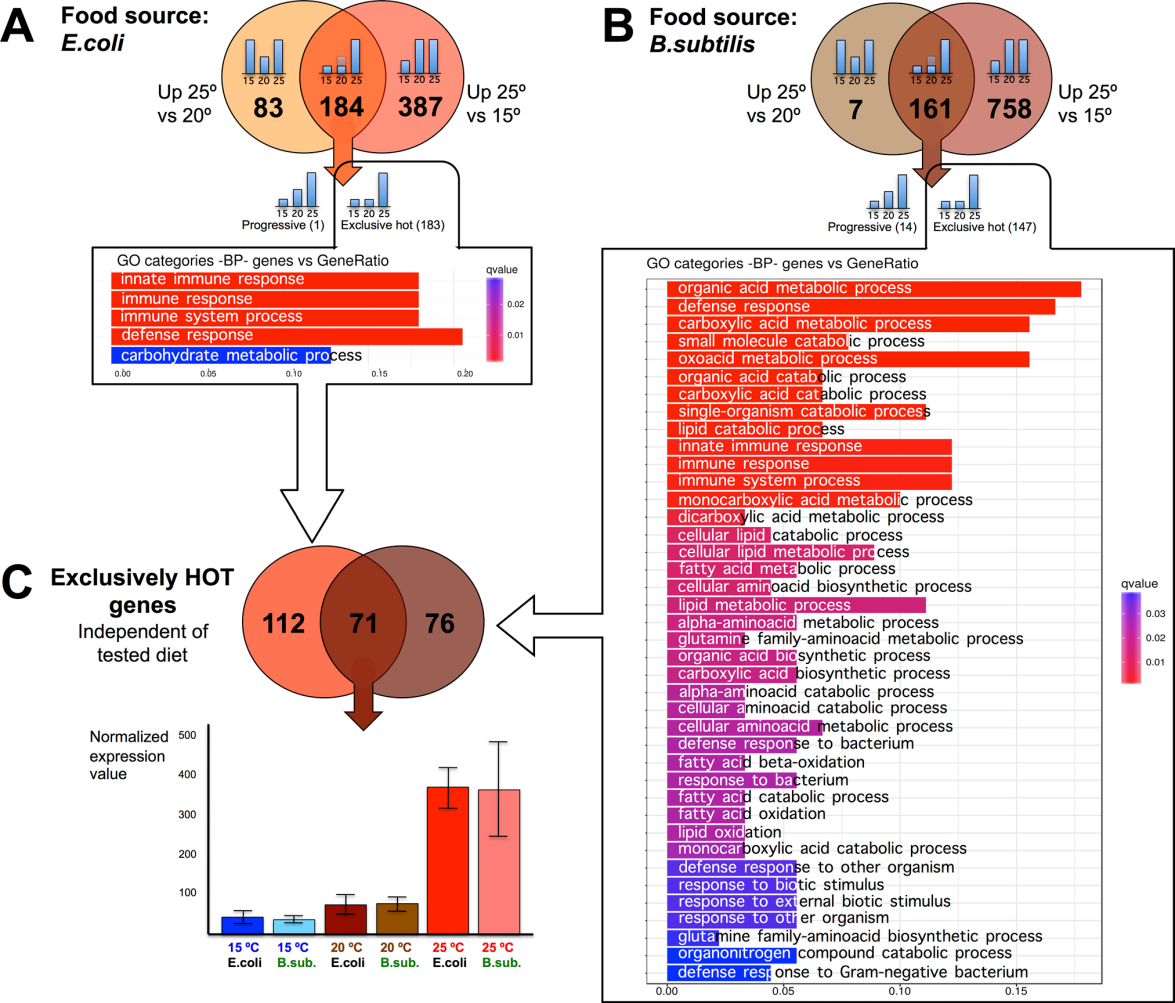
*C. elegans* gene up-regulation at physiologically hot temperature (**A**) Pair comparisons of gene expression in nematodes fed *E.coli* at different temperatures shows that 184 genes exhibit significantly higher expression at 25°C than at 20°C and 15°C. Out of them, 183 genes named “exclusively hot”, kept the same expression level at cold (15°C) or standard growth temperatures (20°C) but up-regulate their expression at physiologically hot temperatures (25°C). This exclusively hot response in worms fed on *E. coli* showed an up-regulation of genes related mainly to defense response pathways. Gene Ontology (GO) Biological Processes (BP) vs number of genes within each category appears represented in color bars, one bar per GO term. The length of the bars indicates the number of genes belonging to the different GO categories and the color indicates the statistical significance, from those with high significant expression difference (red) to those with lower expression difference (blue). (**B**) Pair comparisons of gene expression in nematodes fed *B. subtilis* at different temperatures shows that 161 genes exhibit significantly higher expression at 25°C than at 20°C and 15°C. Out of them, 147 genes named “exclusively hot”, kept the same expression level at cold (15°C) or standard growth temperatures (20°C) but up-regulate their expression at physiologically hot temperatures (25°C). This exclusively hot response in worms fed on *B. subtilis* showed an up-regulation of genes enriched for several functions such as defense response and metabolism of organic acids, lipids and amino acids. Gene Ontology (GO) Biological Processes (BP) vs number of genes within each category appears represented in color bars, one bar per GO term. The length of the bars indicates the number of genes belonging to the different GO categories and the color indicates the statistical significance, from those with high significant expression difference (red) to those with lower expression difference (blue). (**C**) 71 genes show an exclusively hot response independently of the tested diet. However, its Gene Ontology analysis did not show an obvious enrichment for any biological functions beyond the fact of having been up-regulated at 25°C. For the rest of the genes, the exclusive hot response was specific for the *E. coli* diet (112 genes) or specific for the *B. subtilis* diet (76 genes). Expression profile for this set of 71 genes is exemplified in the histogram. There are no significant differences at 15°C or 20°C but a significant upregulation at 25°C, in both diets.

**Figure 4:**
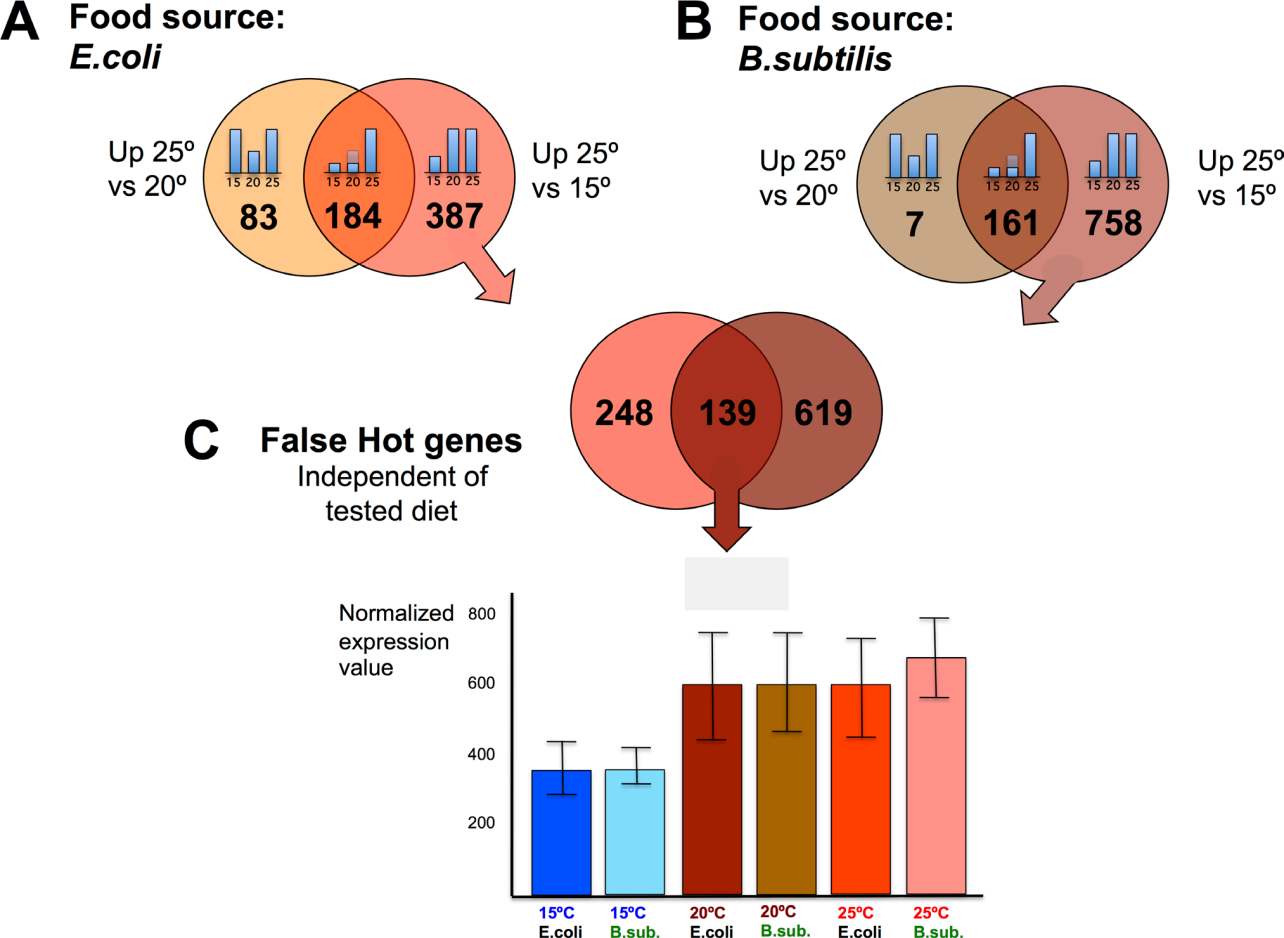
*C. elegans* gene down-regulation at physiologically cold temperature (**A)** *E. coli* fed worms showed 387 genes with significantly higher expression when worms were maintained at 25°C relative to worms maintained at 15°C, but did not show differences relative to worms maintained at 20°C. These 387 genes were referred to as “false hot” genes in an *E. coli* diet. (**B**) *B. subtilis* fed worms showed 758 genes with significantly higher expression when worms were maintained at 25°C relative to worms maintained at 15°C, but did not show differences relative to worms maintained at 20°C. These genes were referred to as “false hot” genes in a *B. subtilis* diet. (**C**) 139 genes behaved as “false hot” genes independently of the tested diet. Expression pattern for this set of genes is exemplified in the histogram. There are no significant differences at 20°C or 25°C but a significant downregulation at 15°C, in both diets.

We wanted to explore the impact on the transcriptome of maintaining *C. elegans* at physiologically high or low temperatures (25°C or 15°C) compared to the standard (20°C) lab growing condition. To do this, we first performed pairwise comparisons of gene expression data sets obtained from worms fed the same diet but maintained at different temperatures. When *C. elegans* were fed the *E. coli* diet, we observed 571 genes with significantly increased expression for worms kept at 25°C compared to worms kept at 15°C. Of these, 184 genes also showed increased expression when worms kept at 25°C were compared to worms kept at 20°C. 183 out of the 184 genes retained the same expression level at 15°C or at 20°C but up-regulated their expression at physiologically hot temperature (25°C). We named these genes “exclusively hot” genes (Figure 3A and Supplementary Table 1). The rest of the genes (387) that showed significantly increased expression when worms were kept at 25°C relative to 15°C, but did not show differences when worms were maintained at 20°C or 25°C, were referred to as “false hot” genes. We observed down-regulation of these “false hot” genes when worms maintained at 15°C were compared to either worms kept at 20°C or at 25°C (Figure 4A and Supplementary Table 2).

The same pairwise comparisons described above were performed for *B. subtilis* fed worms (Figure 3B). When fed this diet, we observed 919 genes with significantly increased expression for worms kept at 25°C compared to worms kept at 15°C. 161 genes of the 919 also showed increased expression when worms were kept at 25°C relative to 20°C. 147 out of the 161 genes kept the same expression level when worms were maintained at 15°C or at 20°C but up-regulated their expression at 25°C. These genes were referred to as “exclusively hot” genes (Figure 3B and Supplementary Table 3). Most of the genes that showed a significantly increased expression when *B. subtilis* fed worms were maintained at 25°C relative to when worms were maintained at 15°C can be considered as “false hot” genes. These genes were down-regulated when worms were kept at 15°C compared to when worms were kept at either 20°C or at 25°C (Figure 4B and Supplementary Table 4).

We analyzed the identity of the “exclusively hot” genes for *E. coli* fed worms and their function by Gene Ontology analysis and observed up-regulation of genes involved in transcriptional activation of defense response pathways and, to a lower extent up-regulation of genes involved in carbohydrate metabolism (Figure 3A). When worms were fed an *E. coli* diet, the “exclusively hot” gene up-regulation was involved in defense response pathways. However, on a *B. subtilis* diet, the “exclusively hot” genes were enriched in genes related to metabolism of organic acids, lipids and amino acids (Figure 3B). We found 71 “exclusively hot” genes whose up-regulation was independent of the diet (Figure 3C and Supplementary Table 5). Gene ontology analysis of these genes did not show an obvious enrichment for any biological functions. However, the presence of a statistically significant (RF: 46.5, *p* < 1.6e-105) common “exclusively hot” gene response suggests the existence of a transcriptional regulatory mechanism to help *C. elegans* adapt to physiologically hot temperatures (25°C).

**Figure 5:**
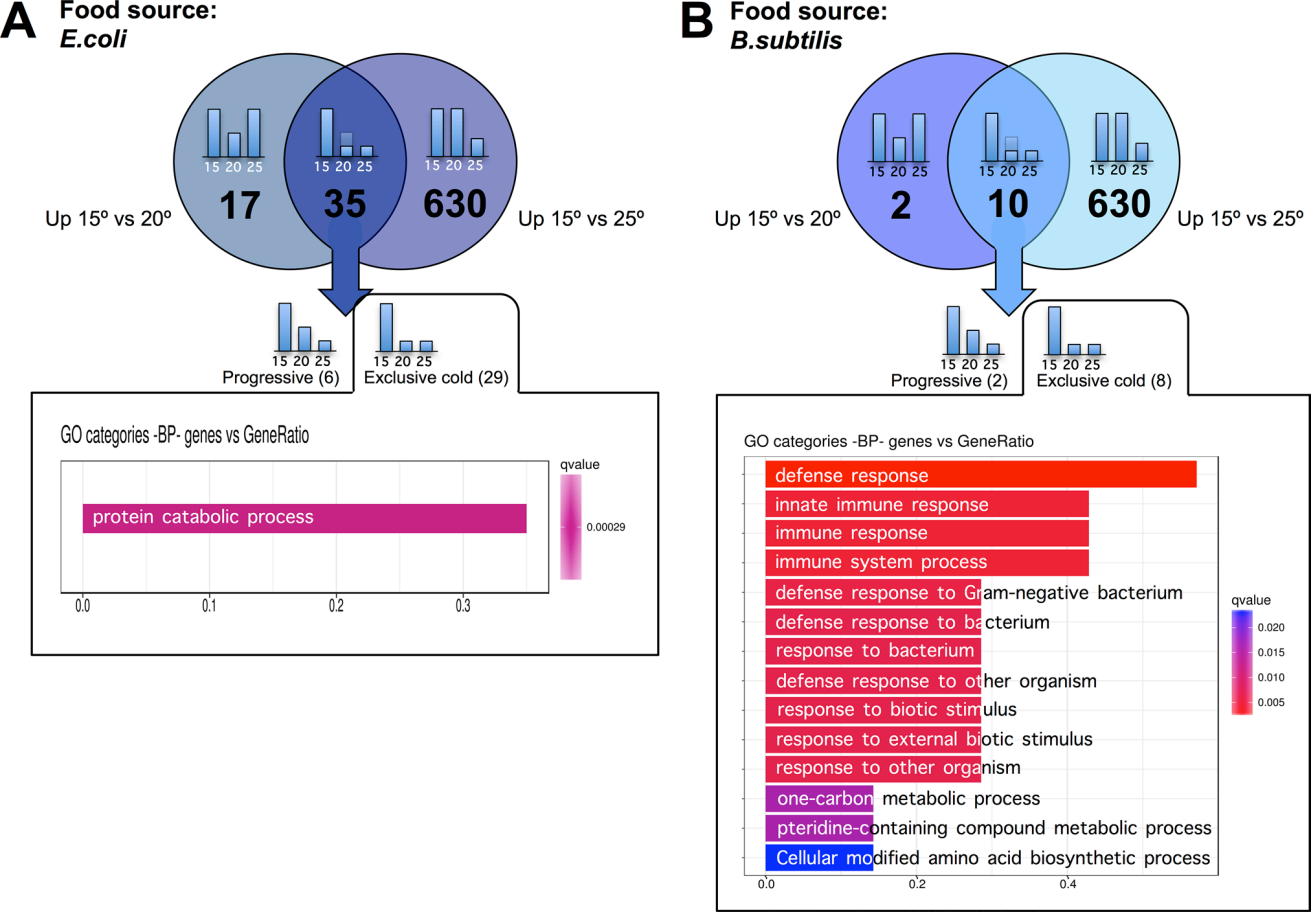
*C. elegans* gene up-regulation at physiologically cold temperature (**A**) *E. coli* fed worms showed 35 genes with significantly higher expression when worms were maintained at 15°C relative to when worms were maintained at 20°C or at 25°C. 29 out of these 35 genes were referred to as “exclusively cold” genes as they kept the same expression level when worms were maintained at 25°C or at 20°C, but up-regulated their expression when worms were maintained at physiologically cold temperature (15°C). The “exclusively cold” response in *E. coli* fed worms showed a mild up-regulation of genes related to protein catabolism processes. Gene Ontology (GO) Biological Processes (BP) versus number of genes within the only statistically significant category is represented in purple bar. The length of the bar correlates to the number of genes belonging to the GO category. (**B**). *B. subtilis* fed worms showed 10 genes with significantly higher expression when worms were maintained at 15°C relative to when worms were maintained at 20°C or at 25°C. 8 out of these 10 genes were referred to as “exclusively cold” genes as they kept the same expression level when worms were maintained at 25°C or at 20°C but up-regulated their expression when worms were maintained at physiologically cold temperature (15°C). The “exclusively cold” response in *B. subtilis* fed worms affects genes involved in defense response. Gene Ontology (GO) Biological Processes (BP) versus number of genes within each category is represented in color bars, one bar per GO term. The length of the bars indicates the number of genes belonging to the different GO categories. The color of the bars indicates the statistical significance, ranging from highly significant expression difference (red) to low expression difference (blue).

**Figure 6:**
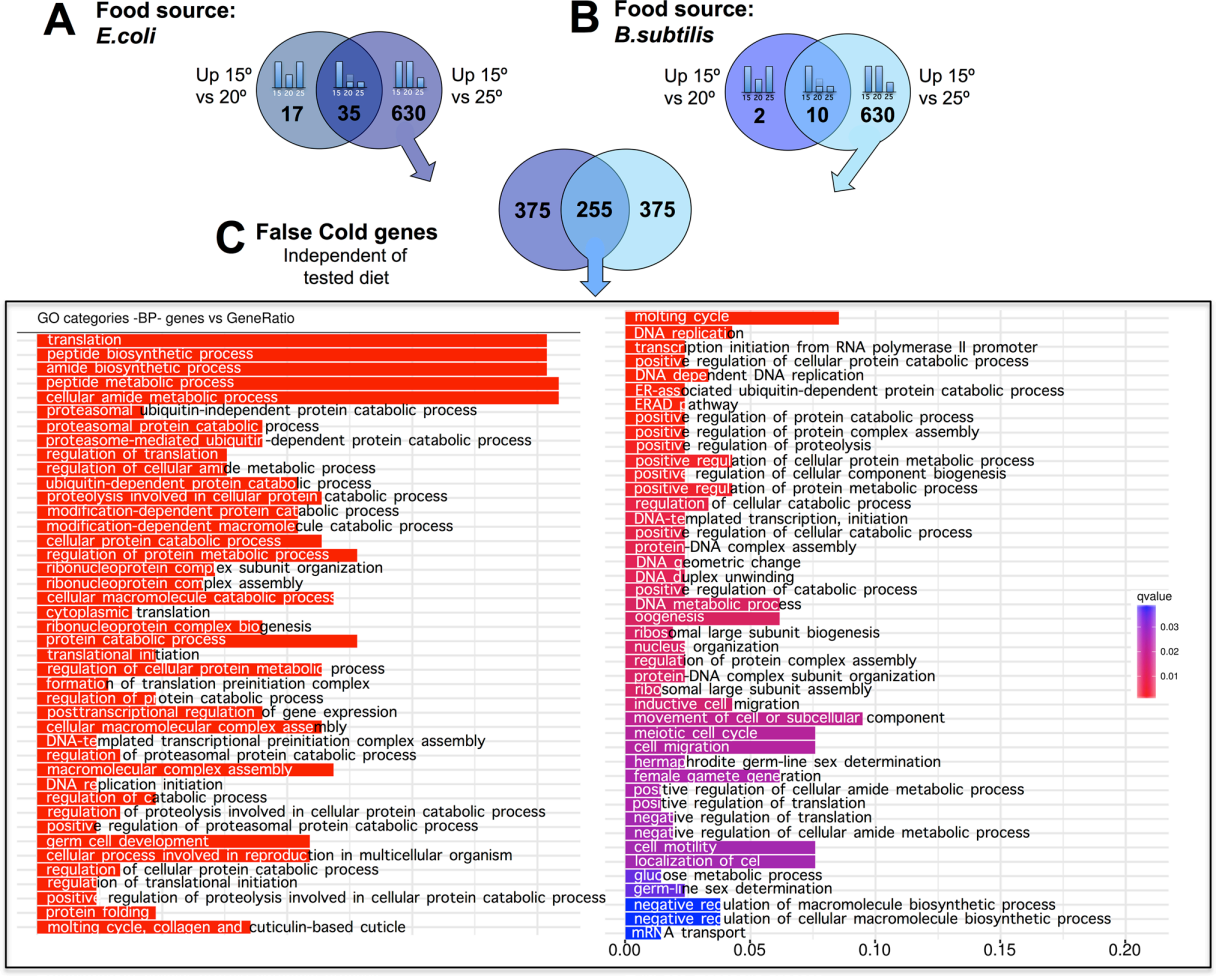
*C. elegans* gene down-regulation at physiologically hot temperature (**A**) *E. coli* fed worms showed 630 genes with significantly lower expression when worms were maintained at 25°C relative to when maintained at 15°C or at 20°C. These genes were referred to as “false cold” genes. (**B)**. *B. subtilis* fed worms showed 630 genes with significantly lower expression when worms were maintained at 25°C relative to when maintained at 15°C or at 20°C. These genes were referred to as “false cold” genes.(**C**). 255 genes showed a “false cold” response independently of the tested diet. These genes are involved in a wide variety of biological processes. Gene Ontology (GO) Biological Processes (BP) versus number of genes within each category is represented in color bars, one bar per GO term. The length of the bars indicates the number of genes belonging to the different GO categories. The color of the bars indicates the statistical significance, ranging from highly significant expression difference (red) to low expression difference (blue).

To characterize the transcriptomic response of worms to physiologically cold temperatures (15°C), we performed similar analyses as the ones outlined above for worms grown at 25°C. When fed an *E. coli* diet, we observed 665 genes with significantly increased expression for worms maintained at 15°C relative to 25°C. 35 of the 665 genes also showed increased expression at 15°C relative to 20°C. 29 of the 35 genes did not show significant differences when worms were maintained at 20°C or at 25°C but up-regulated their expression when the animals were kept at physiologically cold temperature (15°C). We named these genes “exclusively cold” genes (Figure 5A and Supplementary Table 6). The rest of the genes (630) that showed significantly higher expression when worms were maintained at 15°C as compared to 25°C, but did not show differences when maintained at 15°C or at 20°C, were termed “false cold” genes. These 630 genes were observed to be down-regulated when worms were maintained at 25°C compared to when worms were kept at either 20°C or 15°C (Figure 6A and Supplementary Table 7).

The same transcriptomic analyses detailed above were performed when worms were fed *B. subtilis* instead of *E. coli* (Figure 5B). 640 genes showed significantly higher expression when *B. subtilis* fed worms were maintained at 15°C relative to when worms were maintained at 25°C. Of these 640 genes, only 10 also showed increased expression when worms were maintained at 15°C instead of at 20°C. 8 of these 10 genes were referred to as “exclusively cold” genes as they retained the same expression level when worms were kept at 25°C or at 20°C but became up-regulated at physiologically cold temperature (15°C) (Figure 5B and Supplementary Table 8). The vast majority of genes that showed a significantly higher expression when *B. subtilis* fed worms were maintained at 15°C as compared to when maintained at 25°C can be considered “false cold” genes as they were down-regulated in worms kept at 25°C relative to either worms kept at 20°C or at 15°C (Figure 6B, Supplementary Table 9).

Maintaining worms at 15°C on an *E. coli* diet led to a mild activation of protein catabolism genes (Figure 5). However, when worms were maintained at 15°C on the *B. subtilis* diet, we observed a strong activation of genes involved in the defense response (Figure 5). We did not observe a common set of genes whose expression increased in both, *E. coli* and *B. subtilis* fed worms when these were maintained at 15°C (Figure 5). It is important to highlight that the transcriptional activation response that we detected when worms were maintained at physiologically cold temperature (15°C) was much less prominent than that of worms maintained at physiologically hot temperature (25°C) and that the transcriptional response to temperature, either to cold (15°C) or hot (25°C), was strongly dependent on the tested diets.

Analysis of the down-regulated genes when worms were maintained at 25°C, (“false cold” genes) showed a statistically significant (RF: 11.3, *p* < 2.4e-215) common response for *E. coli* and *B. subtilis* fed worms. Functional analysis of the 255 common “false cold” genes reflects enrichment in different aspects of protein metabolism such as synthesis, modification or degradation. In addition, amide metabolism genes showed a significant down-regulation when worms were maintained at 25°C. Other biological processes such as those related to DNA replication or transcription were also down-regulated when worms were kept at 25°C (Figure 6 and Supplementary Table 10). Analysis of “false hot” genes, (genes that are down-regulated when worms were kept at 15°C) also showed a significant common response in both, *E. coli* and *B. subtilis* fed nematodes (RF: 8.3, *p* < 8.6e-92). However, gene down-regulation when worms were kept at 15°C was less prominent than when worms were kept at 25°C (Figure 4 and Supplementary Table 11). Our bioinformatics analyses did not reveal a clear enrichment in any biological processes for the down-regulated genes in worms maintained at 15°C.

Taken together, our results suggest the existence of a transcriptional regulation at physiologically hot (25°C) temperature. This transcriptional response causes both up-regulation as well as down-regulation of specific sets of genes. The transcriptomic response of worms maintained at physiologically cold temperature (15°C) is milder than that of worms maintained at hot temperature (25°C) and is also dependent on the tested diets.

### The *C. elegans* transcriptome is strongly influenced by the diet

**Figure 7:**
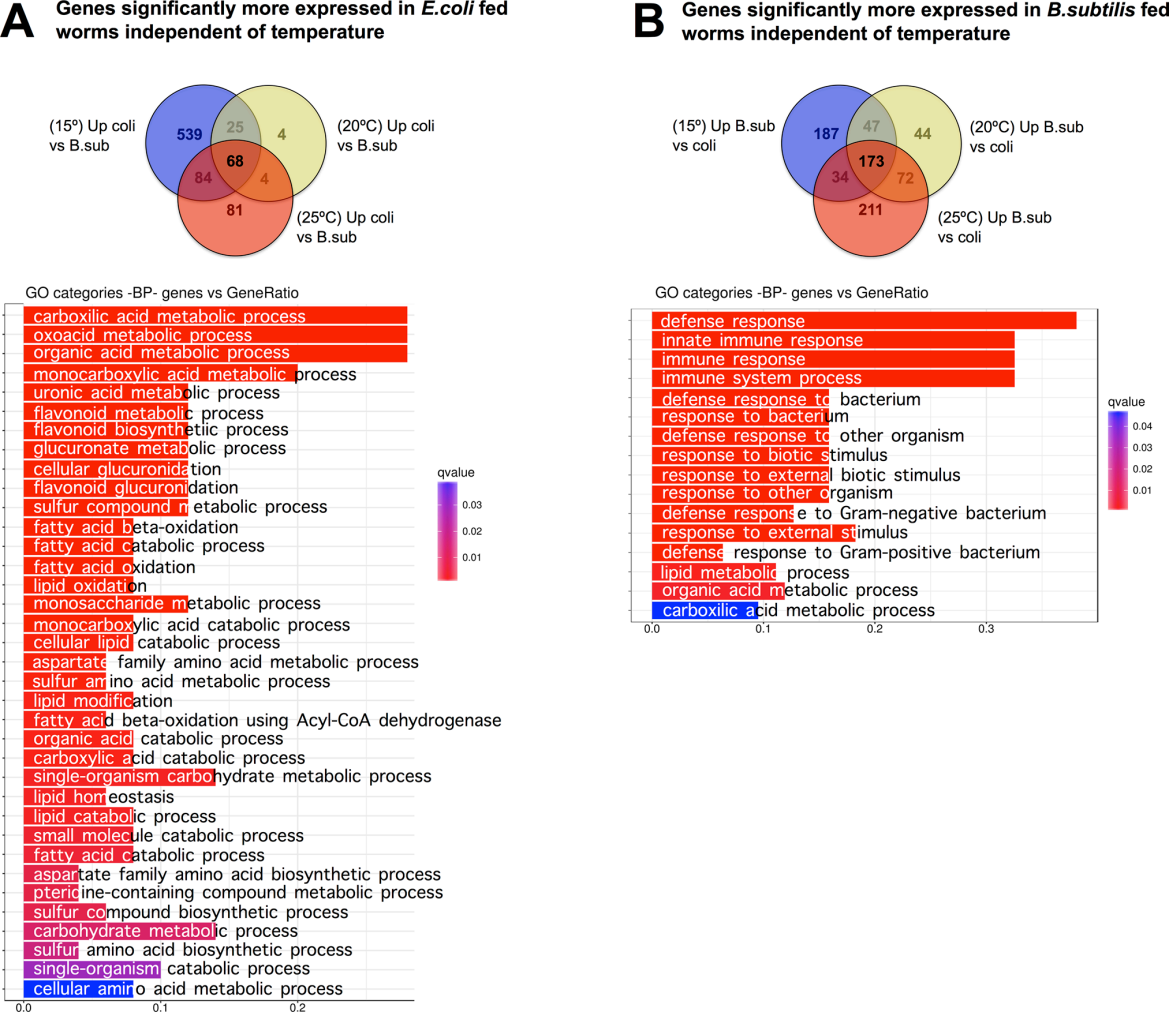
Diet effect on the *C. elegans* transcriptome (**A**) Pairs comparisons of gene expression in nematodes fed each diet at different temperatures shows that 68 genes exhibit significantly higher expression on an *E. coli* diet independently of the growing temperature. Functional analysis of these genes revealed enrichment of biological processes such as: metabolism of organic acids, metabolism of flavonoids, and glucuronidation or lipid metabolism. (**B)** Pairs comparisons of gene expression in nematodes fed each diet at different temperatures shows that 173 genes exhibit significantly higher expression on a *B. subtilis* diet than on an *E. coli* diet, independently of the growing temperature. This group was predominant enrichment in biological defense processes. Gene Ontology (GO) Biological Processes (BP) vs number of genes within each category appears represented in color bars, one bar per GO term. The length of the bars indicates the number of genes belonging to the different GO categories and the color indicates the statistical significance, from those with highly significant expression difference (red) to those with low expression difference (blue).

To understand the effect of the diet (*E. coli* versus *B. subtilis*) on the *C. elegans* transcriptome, we performed pairwise comparisons of gene expression datasets for worms grown at the same temperature on each diet. We identified specific sets of genes that showed a statistically significant higher expression when the nematodes were fed on *E. coli* compared to *B. subtilis* at the three tested temperatures, 15°C, 20°C, and 25°C (Figure 7A and Supplementary Table 12). In the same way, we identified those genes showing a significantly higher expression on a *B. subtilis* diet at the three tested temperatures (Figure 7B, Supplementary Table 13).

Functional analysis of the 68 genes significantly more expressed in worms fed on *E. coli* revealed enrichment of biological process such as metabolism of organic acids, metabolism of flavonoids, glucuronidation or lipid metabolism (Figure 7A). Strikingly, analysis of the 173 significantly more expressed genes in worms fed on *B. subtilis* showed predominant enrichment of biological defense processes (Figure 7B).

## DISCUSSION

Genetic expression of the nematode *C. elegans* in the lab is influenced by a wide variety of factors including the developmental stage of the worms [28] and the chronological age of the animals [30]. Thus, when using *C. elegans* to study biological phenomena, it is key to provide uniformity in the worm growing conditions.

Even though 20°C is considered the standard temperature for growing *C. elegans* in the lab, studies are often performed at other temperatures within the 15°C–25°C worm physiological range. Moreover, the standard diet of *C. elegans* in the lab is the Gram-negative bacterium *E. coli*. However, a number of studies have been published in which the Gram positive bacterium *B. subtilis* is used as an alternative worm diet. Little is known about the global transcriptional response of *C. elegans* to temperature, different bacterial food sources and the crossed response to growth temperature and diet. In our study we wanted to investigate the impact that growing worms at different physiological temperatures (15°C, 20°C, and 25°C) while fed two different diets (*E. coli* and *B. subtilis*) exert on the *C. elegans* transcriptome.

Genome-wide expression analyses have revealed the influence of temperature on processes such as environment-induced plastic response [31] or interactions with toxicants [32]. These studies have been carried out at temperatures in both extremes of the physiological range (∼15°C and ∼25°C) and, together with others, revealed that temperature changes indeed affect the *C. elegans* response to various biological phenomena such as locomotion, chemotaxis, detoxification, metabolism or aging [33–35]. *C. elegans* studies performed at temperatures within the 15°C–25^o^C physiological range thus have the potential to elicit different biological responses by the worm, yet results and conclusions from studies at different temperatures are often compared.

*C. elegans* is a bacteriovore and the standard diet used to maintain worms in the lab is *E. coli*. Recent studies have indicated that a common cause of death for worms grown under normal lab conditions is pathogenicity from the ingested *E. coli* food [8]. In fact, *E. coli* is not a bacterium type that worms typically encounter in the wild [4–5]. The use of *E. coli* as *C. elegans* food responds purely to historical reasons, as *E. coli* was a commonly used and easy to maintain bacterium at the time *C. elegans* was developed as a model organism [2].

In the last few years, different studies have focused on the characterization of wild bacterial food sources and their impact on *C. elegans* metabolism [36–38]. For example, the Gram-positive bacterium *B. subtilis* has been used as an alternative worm diet in a number of studies. While equally nutritious to the worm [11], *E. coli* is mildly pathogenic whereas B. subtilis is not pathogenic [39].

Our transcriptomic analyses did not detect global or massive *C. elegans* gene expression alterations when worms were maintained at 15°C, 20°C, or 25°C. This is not a surprising result as these are temperatures within the *C. elegans* physiological range. However, we detected that the *C. elegans* transcriptomic response to temperature was highly dependent on the *E. coli* and *B. subtilis* diet type.

Gene expression when worms were maintained at 20°C was referred as the control temperature. We considered a gene to be up-regulated at a given temperature when its expression was significantly higher relative to the 20°C control and the other assayed temperature. These genes were grouped as either “exclusively hot” or “exclusively cold” genes. However, we found that for worms maintained at 15°C or at 25°C gene down-regulation was a more prevalent phenomenon than gene up-regulation. Down-regulated genes were grouped as either “false hot” or “false cold” genes. The presence of up- and down-regulated groups of genes suggests that both, transcription induction and well as transcription repression play a role in the adaptation of worms to physiological temperature variations.

We validated the activation/repression of our transcriptomic dataset by RT-qPCR and fluorescent reporters demonstrating that at least some of the temperature responsive genes of our study could be used as *in vivo* “biological thermometer” biomarkers. Thus, genes from our data set might be useful to study genetic pathways involved in the worm adaptation to different environmental temperature conditions.

We found significant differences in the transcription patterns of specific gene sets, which indicate the existence of a transcriptional response to adapt the animal’s metabolism and defense system to the growth temperature. This temperature induced transcriptional response was highly dependent on the bacterial type used as food source. When grown on an *E. coli* diet, *C. elegans* showed a significantly higher expression of genes involved in lipid metabolism, carboxylic acid metabolism, and glucuronidation of different compounds than when worms were grown on the *B. subtilis* diet regardless of the maintenance temperature (Figure 7). This result likely reflects nurturing and detoxification adaptations by *C. elegans* to the particular molecular composition of *E. coli*, a diet that worms are not likely to encounter in the wild [40].

*E. coli* are Gram-negative bacteria. Thus, the *E. coli* cell envelope is essentially composed of an outer lipid bilayer membrane, a peptidoglycan cell wall and another inner lipid bilayer membrane [41]. *B. subtilis* are Gram-positive bacteria, consequently they lack the outer lipid membrane [42]. Despite cell structure differences, *B. subtilis* and *E. coli* have been reported to have a similar caloric content as the main calorie contribution is conferred by proteins and carbohydrates and not by the lipid fraction [11]. It is however plausible that the different lipid composition and distribution found in these two types of bacteria, 80% of phosphatidylethanolamine in the membrane lipids of *E. coli* versus 12% in *B. subtilis* [40], causes up-regulation of specific sets of genes required for *E. coli* digestion and metabolism of its components such as carboxylic acids.

In addition to lipid metabolism, when fed an *E. coli* diet, *C. elegans* enhances expression of genes involved in glucuronidation of specific substrates. Glucuronidation is driven by *ugt* (UDP Glycosyltransferase) genes (Figure 7). These genes encode enzymes that catalyze the linkage of glucuronic acid to specific substrates. The glucuronic acid group modifies the substrate properties and terminates their biological actions by promoting their elimination. Therefore, glucuronidation is involved in detoxification of compounds and metabolites that cause a detrimental effect to the organism [43]. Among the substrates of UGTs is the quinone Coenzyme Q (CoQ). CoQ and its metabolites play fundamental redox roles in living organisms such as being a crucial mitochondrial electron transport chain carrier or conferring antioxidant protection to cell membrane components [44–45]. However, excessive CoQ redox activity leads to toxic effects thus CoQ must be maintained at physiological levels by balancing its rate of synthesis and degradation [46–47]. Interestingly, *C. elegans* and *E. coli,* but not *B. subtilis,* synthesize their own coQ [11]. Thus, when *C. elegans* are fed an *E. coli* diet, worms uptake additional CoQ produced by the bacterial diet [48]. In contrast, *B. subtilis* does not synthesize CoQ. Accordingly, when we maintained *C. elegans* on a *B. subtilis* diet we did not observe activation of pathways involved in CoQ elimination.

*B. subtilis* fed worms live substantially longer than worms grown on *E. coli* [6] and these diets appear to induce different causes of death to the worms [8, 49]. It has been recently proposed that the *C. elegans* life span difference observed when the worm diet is changed from the standard *E. coli* to *B. subtilis* is mainly due to the persistent antioxidant effect of CoQ present in the *E. coli* diet, which leads to an imbalance in cellular REDOX homeostasis [11]. Feeding worms *E. coli* or *B. subtilis* diets can considerably affect the life span of the worms, consequently *E. coli* fed worms will display an older physiological age than *B. subtilis* fed worms with the same chronological age. To minimize this issue, all our transcriptomic comparisons were performed using synchronized adult nematodes of very young chronological age (1 day old).

As stated above, *B. subtilis* is a less pathogenic diet than *E. coli* and worms fed this diet live longer than *E. coli* fed worms [6]. A counterintuitive observation arising from our transcriptomic analysis is the higher expression of genes involved in the defense response when worms were fed *B. subtilis* instead of *E. coli* (Figure 7). *B. subtilis* cells sporulate while *E. coli* do not. *B. subtilis* spores are not digestible, thus it is possible that *B. subtilis* spores induce up-regulation of defense mechanism pathways by *C. elegans*. Moreover, *B. subtilis* may provide factors involved in molecular signaling not present in *E. coli*, which could contribute to up-regulation of stress resistance and defense mechanisms. An example of such a factor is nitric oxide. Nitric oxide is produced by *B. subtilis* but not by *E. coli*. *C. elegans* lack the enzymes necessary to produce their own nitric oxide. Gusarov et al. showed that nitric oxide signaling upon *B. subtilis* feeding leads to enhanced *C. elegans* longevity and stress resistance [50]. These signaling differences upon *B. subtilis* feeding could also explain the fact that genetic and environmental interventions that affect *C. elegans* longevity by affecting different molecular pathways do not always lead to the same effects when worms are fed *E. coli* or *B. subtilis* [11].

**Figure 8:**
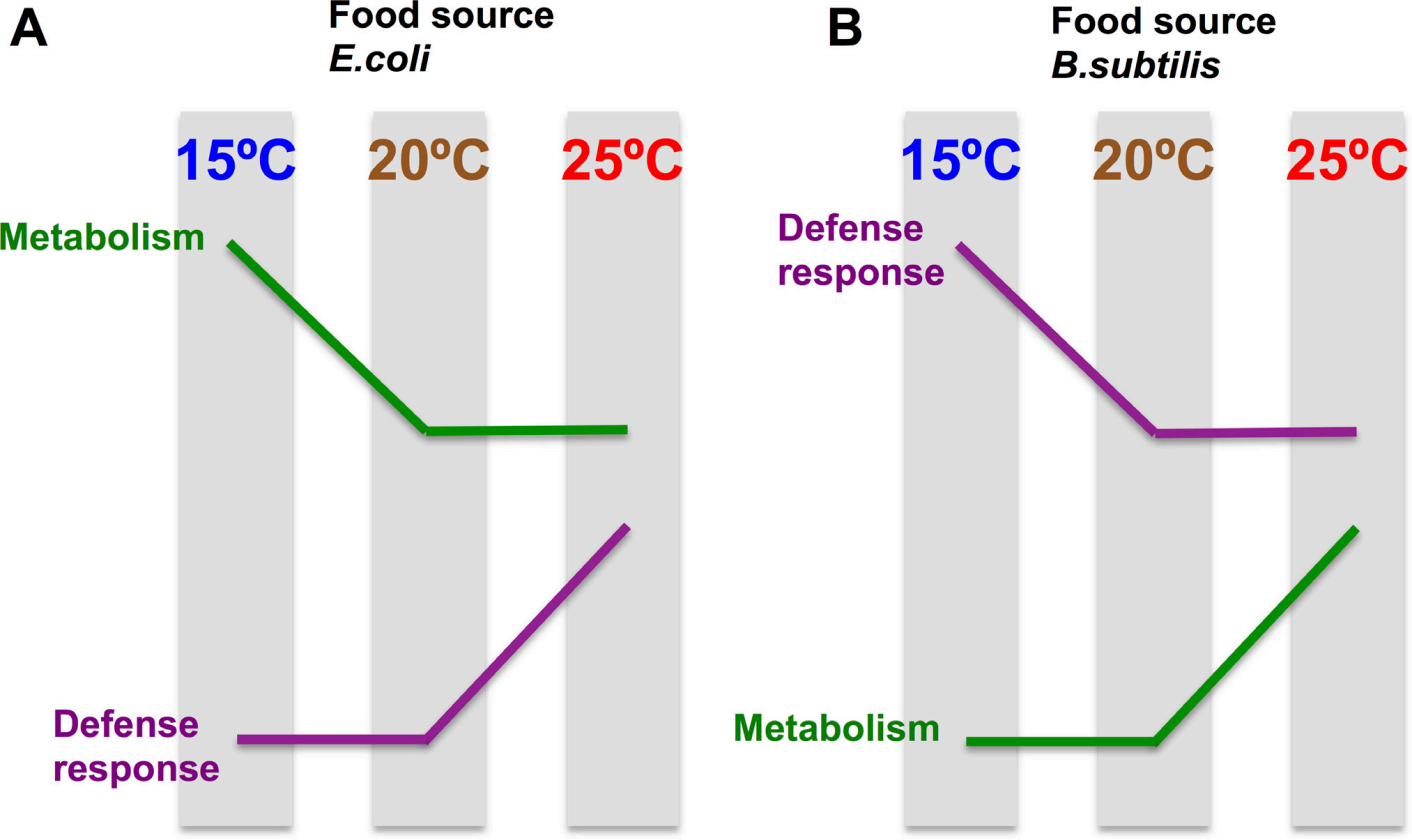
The transcriptomic temperature response is opposite in nematodes grown on an *E. coli* diet vs a *B. subtilis* diet (**A**) *E. coli* fed worms enhance defense response gene expression and decrease expression of genes associated with metabolic functions when the maintenance temperature increases. Genes involved in metabolism are significantly up-regulated in *E. coli* fed worms compared to *B. subtilis* fed worms, independently of the tested temperature. (**B**). *B. subtilis* fed worms decrease defense response gene expression and enhance expression of genes associated with metabolic functions when the maintenance temperature increases. Genes involved in defense response are significantly up-regulated in *B. subtilis* fed worms compared to *E. coli* fed worms, independently of the tested temperature.

Analysis of Motif Enrichment (AME) (http://meme-suite.org) of the promoter region of the 71 “exclusively hot” genes (activated at 25°C on both diets) reveals enrichment on motifs recognized by the MDL-1 and HLH-15 (Supplementary Figure 1). This suggests that transcriptional regulation at this temperature might be mediated by the action of these transcription factors. MDL-1 and HLH-15 are both helix-loop-helix transcription factors and have been described to have regulatory functions in processes such as pathogen defense [51], longevity [52–53] and metabolism [54]. Interestingly, the transcriptomic response of worms maintained at different temperatures is the opposite for *E. coli* fed worms compared to *B. subtilis* fed worms. When worms are grown on *E. coli* and the temperature is increased to 25°C, we observed an enhancement of defense response gene expression and decreased expression of genes associated with metabolic functions. However, when worms are grown on *B. subtilis* and the temperature is increased to 25°C, *C. elegans* decreased defense response gene expression and enhanced expression of genes associated with metabolic functions (Figure 8). These results indicate that the effect of the diet on the worm transcriptome can be modulated by fluctuations in the physiological temperature range of the worm.

The worm transcriptional response to temperature might also be influenced by the growth efficiency of the bacterial diet. For example, the optimal growth temperature for bacteria is higher than that of *C. elegans*: 37°C for *E. coli* [55], and 28–35°C for *B. subtilis* (www.atcc.org) compared to 20°C for *C. elegans*. Therefore, when worms are grown at 25°C, the temperature is closer to the optimal temperature for bacterial growth than at 20°C or at 15°C. This could explain why when *C. elegans* are maintained at a higher temperature in the more pathogenic *E. coli* diet, the nematode activates transcription of genes involved in the immune defense system. Under these adverse conditions metabolism is down-regulated. In contrast, when worms are grown on a non-pathogenic diet, optimal growth conditions for the bacterial food source correlate with an up-regulation of genes involved in metabolism to ensure proper nutrition and growth.

In summary, we report that *C. elegans* undergo significant transcriptomic changes when maintenance temperature fluctuates between the physiologically accepted experimental range. Our data also show that the *C. elegans* transcriptional response to temperature highly depends on the bacterium type used as lab diet. Altogether, our results suggest an adaptive mechanism of the nematode metabolism to the different composition and toxicity of the food sources and temperature growth conditions. This mechanism might be conserved through evolution and would ensure animal survival in a changing environment.

## MATERIALS AND METHODS

### Strains and growth conditions

Wild-type (N2) nematodes and the transgenic strain AY101: cIs101 [F35E12.5p::GFP + *rol-6*(*su1006*)] were obtained from the *Caenorhabditis* Genetic Center (CGC, University of Minnesota) and immediately stored as frozen stocks to minimize the genetic drift that may happen in isolated populations growing along the time [12]. Worms were thawed and grown under standard conditions (http://www.wormbook.org) on NGM plates seeded with *E. coli* OP50 or *B. subtilis* PY79 as food source, and incubated at 15°C, 20°C and 25°C during at least 3 generations before starting the experiments. After that, worm populations were synchronized by “bleaching” with hypochlorite solution, maintained in M9 medium during 20 hours to allow hatching eggs and synchronization at the first larval stage (L1) [13]. Then, worms were grown at the corresponding temperatures and bacteria diet until day 1 of adulthood, at the beginning of egg laying (120h at 15°C, 84h at 20°C, 60h at 25°C). To conduct experiments, 5–7 ml of distilled water was poured on to each plate of worms and gently swirled for 10 seconds to wash the worms off the plate. The water containing the worms was then transferred to a 50 ml centrifuge tube and the worms were allowed to settle to the bottom of the tube. The worms were washed with fresh distilled water 2–3 times to remove bacteria and other food sources. The worm sediment was used for the subsequent RNA extraction. Experiments were performed in triplicate to provide a statistical value for gene expression comparison between the different conditions (Figure 1A).

### RNA extraction

RNA from *C.elegans* cultured at 15°C, 20°C and 25°C feeding *E. coli* OP50 or *B. subtilis* PY79 diet was isolated using an RNeasy Mini kit and treated with DNase I (Qiagen) following the manufacturer’s instructions. Integrity and concentration of RNA was determined using the Experion^™^ automated electrophoresis system (Bio-Rad). RNA with an integrity value > 8.9 underwent further analysis.

### cDNA library preparation and ultrasequencing

Sequencing libraries were prepared by following the TruSeq RNA Library (LS) Preparation Kit v2 (Illumina Inc.) from 500 ng of mRNA. All libraries were run in a HiSeq 1500 PE100 lane in Rapid mode, pooled in equimolar amounts to a 10 nM final concentration. Library concentration was measured via qPCR using the KAPA library quantification kit for Illumina sequencing platforms (Kapa Biosystems) before high throughput sequencing.

### Validation of the sequencing results

Gene products with significant expression differences at 15°C and 25°C (T05E11.9, *nex-1* and D2045.2) underwent further confirmation by Real-Time PCR. 500 ng from each RNA extraction was DNAse treated (Promega) and reverse transcribed with SuperScript III First-Strand Synthesis kit (Invitrogen) in a total volume of 20 microlitres according to the manufacturer’s instructions. The mRNA quantification was performed by Real Time-PCR using Power SYBR Green Master Mix (ThermoFisher Scientific) and specific primers in a 7300 quantitative Real Time PCR System (Applied Biosystems, Carlsbad, CA). Normalization to actins’ expression (*act-1*) was used to calculate relative expression. The experiments were repeated in three independent replicas. Primers pairs used for RT-qPCR were: T05E11.9 Fw: TTTGGAGTGCCCCTGTATGT; T05E11.9 Rv: ATGCTCTTCCAACCAGACGA; ZC155.1 Fw: TGTCAACGTCATCACCTCGA; ZC155.1 Rv: TCCCTTCATGGCAGCCTTAA;D2045.2 Fw: GGAAGCGATTCTCCGTGTTC; D2045.2 Rv: CAACGACAGGCAGAATCTCG*act-1* Fw: CCGAGCGTGGTTACTCTTTC; *act-1* Rv: GCGATTTCTTGCTCGAAGTC.

### Bioinformatic analysis

The quality of RNA-seq results was firstly assessed using FastQC (http://www.bioinformatics.babraham.ac.uk/projects/fastqc/). The raw reads were trimmed, filtered with a Phred quality score of at least 25 and all adapters removed with Trimmomatic software [14].

Clean reads were aligned versus the N2 *Caernohabditis elegans* reference genome (release WBcel235.85, http://www.ensembl.org/Caenorhabditis_elegans/Info/Index) by using Tophat2 [15] with default parameters. Resulting alignment files were quality assessed with Qualimap2 [16] and sorted and indexed with Samtools software [17]. After read count on gene features with the FeatureCounts tool [18], quantitative differential expression analysis between conditions was performed both by DESeq2 [19] and edgeR [20] implementations, to compare all groups in pairs to analyze the effect of the two variables of this study: food sources (*E. coli* diet vs *B. subtilis* diet) and growth temperatures (15°C vs 20°C vs 25°C). Both of them, implemented as R Bioconductor packages, perform read-count normalization by following a negative binomial distribution model. In order to automate this process and facilitate all group combination analysis, the SARTools pipeline [21] was used. The resulting data was obtained as an .html file and .csv tables, including density count distribution analysis, pairwise scatter plots, cluster dendrograms, Principal Component Analysis (PCoA) plots, size factor estimations, dispersion plots and MA- and volcano plots. Resulting tables, including raw counts, normalized counts, Fold-Change estimation and dispersion data for each of the analysis methods (DESeq2 and edgeR) were annotated with additional data from Biomart (https://bioconductor.org/packages/release/bioc/html/biomaRt.html), WormBase (http://www.wormbase.org) and org.Ce.eg.db (https://bioconductor.org/packages/release/data/annotation/html/org.Ce.eg.db.html) databases. Final tables also include the associated gene name, Ensembl Transcript and protein information, GO Term ID and names, EntrezID, UniprotTrEMBL information and Human ortolog ID and gene name data. Normalized values for each gene were graphically represented into barplots for all samples under different temperature and feeding conditions.

To control the False Discovery Rate (FDR), p-values were amended by Benjamini-Hochberg (BH) multiple testing correction [22]. Those features showing corrected p-values below a 0.05 threshold were considered as significantly up- or down-regulated genes. In order to reinforce downstream analysis and discard false-positive over/under-expressed genes, common up- and down-regulated features were extracted from DESeq2 and edgeR tables. The CRAN packages eVenn (https://CRAN.R-project.org/package = eVenn) and pheatmap (https://CRAN.R-project.org/package = pheatmap) were used to graphically represent Venn diagrams and heatmap plots showing these common features.

Gene Ontology enrichment analysis was performed for common up/down regulated genes by using clusterProfiler package [23] through its enrichGO tool. This tool uses a hypergeometric BH model to obtain adjusted q-values. Each GO category (Biological Process –BP-, Molecular Function –MF, and Cellular Component –CC-) was represented in barplots, showing its relative abundance and associated *q*-value. Similarly, KEGG and Reactome pathway analysis was conducted by clusterProfiler (enrichKEGG) and ReactomePA [24] tools. The KEGG pathway maps were obtained with Pathview package [25].

The regularized log2 transformation (rlog) data was represented into a 2-dimension Principal Component Analysis (PCA) plot, by tacking DESeq2 expression values (Figure 1B). It shows temperature growing conditions in different colors and food types in different plotting symbols. Sample-to-sample Euclidean distances were calculated from the rld data and represented in a heatmap, showing the adjacent clustering information (Figure 1C).

### Statistical analysis

Statistical significance of the overlap between groups of genes was assessed by calculation of the Representation Factor. The Representation Factor (RF) is the number of overlapping genes divided by the expected number of overlapping genes drawn from two independent groups. A representation factor > 1 indicates more overlap than expected of two independent groups; a representation factor < 1 indicates less overlap than expected; and a representation factor of 1 indicates that the two groups overlap by the number of genes expected for independent groups of genes (http://nemates.org/MA/progs/overlap_stats.html).

## SUPPLEMENTARY MATERIALS FIGURE AND TABLES




























