# Supplementary material for: Effect of the diet type and temperature on the C. elegans transcriptome
Source: Oncotarget. 2017 Dec 21;9(11):9556–71. doi: 10.18632/oncotarget.23563 (PMC5839384; doi:10.18632/oncotarget.23563)
Supplement: Supplementary file 1 [file oncotarget-09-9556-s001.pdf]

Effect of the diet type and temperature on the *C. elegans* transcriptome

SUPPLEMENTARY MATERIALS

Sequence	Uniprobe ID	Name	P-value
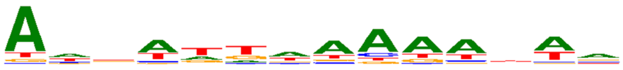	UP00382 1	MDL-1	5.30e-4
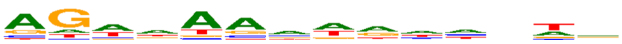	UP00361 1	HLH-15	1.46e-3

Supplementary Figure 1: Analysis of Motif Enrichment (AME) of the promoter regions of the 71 exclusively hot genes independently of the tested diet shows enrichment on motifs recognized by MDL-1 and HLH-15 transcription factors. Sequence indicates the motif identified in the promoter of the 71 “exclusively hot” genes. Uniprobe ID and name identify the transcription factor that recognize and bind these sequences. *P*-value indicates the statistical enrichment of this motif.

Supplementary Table 1: List of exclusively hot genes in *E. coli fed worms.* Show genes that are significantly upregulated at 25ºC compared to both 15ºC and 20ºC, with no statistical difference between 15ºC and 20ºC, in worms feeding ***E. coli***. See Supplementary_Table_1

Supplementary Table 2: List of false hot genes in *E. coli fed worms* (downregulated at 15ºC). Show genes that are significantly downregulated at 15ºC compared to both 20ºC and 25ºC, with no statistical difference between 20ºC and 25ºC, in worms feeding *E. coli*. See Supplementary_Table_2

Supplementary Table 3: List of exclusively hot genes in *B. subtilis fed worms.* Show genes that are significantly upregulated at 25ºC compared to both 15ºC and 20ºC, with no statistical difference between 15ºC and 20ºC, in worms feeding *B. subtilis*. See Supplementary_Table_3

Supplementary Table 4: List of false hot genes in *B. subtilis fed worms* (downregulated at 15ºC). Show genes that are significantly downregulated at 15ºC compared to both 20ºC and 25ºC, with no statistical difference between 20ºC and 25ºC, in worms feeding *B. subtilis*.Supplementary Table 5 List of common exclusively hot genes in *E. coli* and *B. subtilis*.Show common genes that are significantly upregulated at 25ºC compared to both 15ºC and 20ºC, with no statistical difference between 15ºC and 20ºC, in worms feeding either *E. coli* or *B. subtilis*. See Supplementary_Table_4

Supplementary Table 5: List of common exclusively hot genes in *E. coli* and ***B. subtilis fed worms.*** Show common genes that are significantly upregulated at 25ºC compared to both 15ºC and 20ºC, with no statistical difference between 15ºC and 20ºC, in worms feeding either E. coli or B. subtilis.See Supplementary_Table_5

Supplementary Table 6: List of exclusively cold genes in *E. coli fed worms.* Show genes that are significantly upregulated at 15ºC compared to both 20ºC and 25ºC, with no statistical difference between 20ºC and 25ºC, in worms feeding *E. coli*. See Supplementary_Table_6

Supplementary Table 7: List of false cold genes in *E. coli fed worms* (downregulated at 25ºC). Show genes that are significantly downregulated at 25ºC compared to both 15ºC and 20ºC, with no statistical difference between 15ºC and 20ºC, in worms feeding *E. coli*. See Supplementary_Table_7

Supplementary Table 8: List of exclusively cold genes in *B. subtilis fed worms.* Show genes that are significantly upregulated at 15ºC compared to both 20ºC and 25ºC, with no statistical difference between 20ºC and 25ºC, in worms feeding *B. subtilis*. See Supplementary_Table_8

Supplementary Table 9: List of false cold genes in *B. subtilis fed worms* (**downregulated at 25ºC)**. Show genes that are significantly downregulated at 25ºC compared to both 15ºC and 20ºC, with no statistical difference between 15ºC and 20ºC, in worms feeding *B. subtilis*.See Supplementary_Table_9

Supplementary Table 10: List of common false cold genes in *E. coli* and ***B. subtilis fed worms.*** Show common genes that are significantly downregulated at 25ºC compared to both 15ºC and 20ºC, with no statistical difference between 15ºC and 20ºC, in worms feeding either *E. coli* or *B. subtilis*. See Supplementary_Table_10

Supplementary Table 11: List of common false hot genes in *E. coli* and ***B. subtilis fed worms.*** Show common genes that are significantly downregulated at 15ºC compared to both 20ºC and 25ºC, with no statistical difference between 20ºC and 25ºC, in worms feeding either *E. coli* or *B. subtilis*. See Supplementary_Table_11

Supplementary Table 12: List of genes upregulated on an *E. coli* diet independently of the temperature. Show genes that have a significantly higher expression in worms feeding *E. coli* than in worms feeding *B. subtilis*, independently of the tested temperature (15, 20 or 25ºC). See Supplementary_Table_12

Supplementary Table 13: List of genes upregulated on a *B. subtilis* diet independently of the temperature. Show genes that have a significantly higher expression in worms feeding *B. subtilis* than in worms feeding *E. coli*, independently of the tested temperature (15, 20 or 25ºC). See Supplementary_Table_13
